# Impact of increased numbers of intensive care consultants on outcome in a central London teaching hospital

**DOI:** 10.1186/cc9893

**Published:** 2011-03-11

**Authors:** V Metaxa, C Bell, A Feehan, K Peters, T Chang, P Hopkins, M Ervine

**Affiliations:** 1 King's College Hospital, London, UK

## Introduction

The standardised mortality ratio (SMR) is a key parameter by which ICUs quantify their performance. We report the effect of increased ICU consultant numbers on mortality and SMR in a central London teaching hospital.

## Methods

The study was registered with the Clinical Audit Support System. Data were collected prospectively from March 2005 to date by a dedicated audit team and were analysed as part of routine audit.

## Results

Table 1 shows the reduction in mortality and SMR from 2005 to date, comparing these data with patient and consultant numbers.

**Table 1:** SMR and mortality reduction from 2005 to date

	Number of admissions	Number of consultants	Median APACHE II	ICU mortality (%)	ICU SMR
2005 to 2006	646	5	18	34.4	0.93
2006 to 2007	774	5	17	19.3	1.15
2007 to 2008	842	9	14	16.8	0.91
2008 to 2009	1,510	13	14.5	14.7	0.77
2009 to 2010	1,671	13	17	19.3	0.67
2010 to 2011 (incomplete)	1,434	17	19	19	0.62

## Conclusions

In this study we describe the remarkable reduction in both mortality and SMR that has occurred in the general ICU at King's College Hospital over the past 5 years. The improvement in outcomes was associated with a quadrupling of ICU consultant numbers. We hypothesize that this increase in intensivist numbers allowed the reinforcement of a closed model of ICU care. We are now further analysing these data to search for quantitative improvements in surrogate markers of quality of care over the same time frame.
